# Evaluating the effects of dance on motor outcomes, non-motor outcomes, and quality of life in people living with Parkinson’s: a feasibility study

**DOI:** 10.1186/s40814-022-00982-9

**Published:** 2022-02-09

**Authors:** Anna M. Carapellotti, Matthew Rodger, Michail Doumas

**Affiliations:** School of Psychology, Queen’s University Belfast, 18-30 Malone Road, Belfast, BT9 5BN UK

**Keywords:** Parkinson’s disease, Dance, Quality of life, Feasibility study

## Abstract

**Background:**

Community-based dance programs for people living with Parkinson’s have grown in popularity over the past two decades. Studies investigating these programs have demonstrated multidimensional benefits in motor, non-motor, and quality of life related outcomes, yet there is a need to focus on the feasibility of larger trials. The primary objective of this study was to assess the feasibility and acceptability of conducting a trial investigating dance and Parkinson’s in Northern Ireland. The secondary objectives were to conduct preliminary analyses of the classes’ effects and to assess the appropriateness of outcome measures for a randomized controlled trial.

**Methods:**

Participants were recruited through the community, Parkinson’s UK, and university contacts to participate in a 12-week dance intervention inspired by the Dance for PD® model. Pre- and post-intervention, participants completed the following outcomes: MDS-UPDRS III, TUG, DT-TUG, Sensory Organization Test, MoCA, Trail Making Tests A&B, Digit Symbol Substitution Test, Digit Span, PDQ-39, FOG-Q, PHQ-9, FES-I, and an exit questionnaire (post-test only). Data were analyzed using paired samples *t* tests or Wilcoxon signed ranked test.

**Results:**

Ten people living with Parkinson’s participated. Running a larger trial was deemed infeasible in this setting due to recruitment issues; conversely, the dance intervention was accepted by participants with all but one completing the study. Functional mobility (TUG), symptoms of depression (PHQ-9), and bodily discomfort showed improvement. All other outcomes did not. The exit questionnaire revealed that the social aspect of classes was important, and improvements in mood or mental state were cited most frequently as perceived benefits. Outcome measures were feasible, with some changes suggested for future trials.

**Conclusions:**

This study highlighted the infeasibility of running a larger trial using this design in this setting despite demonstrating the acceptability of implementing a dance program in Northern Ireland for people living with Parkinson’s. The results support existing evidence demonstrating that dance may improve functional mobility and symptoms of depression in people living with Parkinson’s, though the study design and small sample size prevent the generalizability of results. The findings also support the idea that dancing has the potential to support several aspects of physical, emotional, mental, and social health.

**Supplementary Information:**

The online version contains supplementary material available at 10.1186/s40814-022-00982-9.

## Key messages regarding feasibility

- What uncertainties existed regarding the feasibility?
  This study assessed the acceptability of implementing a dance program based on the Dance for PD® model in Northern Ireland and the feasibility of conducting a properly powered trial in this context.
- What are the key feasibility findings?
  The recruitment of participants living with Parkinson’s was a challenge, with only 33% of target reached. Retention of those recruited on the contrary was extremely high with all but one participant completing 20 classes within 12 weeks and the post-test assessments, highlighting the acceptability of the intervention among those who took part.
- What are the implications of the feasibility findings for the design of the main study?
  Given the findings of our study and the small samples in existing research, networked research efforts may be required to recruit the number of participants needed to robustly test the efficacy of dance using an RCT design.

## Introduction

Parkinson’s is a complex neurological condition characterized by motor and non-motor symptoms that negatively impact quality of life (QOL) [1]. The primary motor symptoms of Parkinson’s include bradykinesia, tremor, rigidity, and postural instability [2]. Non-motor symptoms, such as mental health issues (e.g., depression), cognitive impairment, pain, and fatigue, may have a greater impact on QOL than motor symptoms in people living with Parkinson’s and are therefore increasingly recognized as important to manage [3]. Activities of daily living, particularly those that are dependent on walking, can become increasingly challenging as the condition progresses [4], consequently leading to decreased independence and participation [5]. Traditional medical treatments can alleviate symptoms to a certain extent; however, these methods do not fully address balance problems [6], non-motor symptoms [7], stigma [8], pain [9, 10], and the complex nature of living with a chronic, progressive condition. There is thus a need for complementary therapeutic strategies that support people living with Parkinson’s and aim to improve QOL.

Dance is one activity that has been demonstrated to improve both motor and non-motor symptoms [11], offer social support [12, 13], and increase activity participation [14] in people living with Parkinson’s. Community-based dance programs specifically offered to people living with Parkinson’s have been growing in popularity over the past two decades [15] and may offer multidimensional benefits [11]. The Dance for PD® model, for example, has had worldwide success at being implemented in more than 300 communities since 2001 [16], and a number of preliminary studies have been conducted to evaluate its effects [11, 13, 15, 17–21].

In the first evaluation of Dance for PD®, participants most often cited “participating in active recreation,” “socializing,” and “health” when asked what areas of their lives had “changed for the better” as a result of taking part [15]. Since this preliminary evaluation, several studies have investigated the effects of Dance for PD® style classes on QOL and motor and non-motor symptoms. The studies evaluating changes in QOL using questionnaires like the Oregon Health & Sciences University Quality of Life Scale (QOLS) and Parkinson’s Disease Questionnaire (PDQ-39) have demonstrated inconsistent results, with some studies reporting improvements [17, 21] and others no change [13, 18, 20]. In the evaluation of motor symptoms, Westheimer and colleagues [13] found the Movement Disorder Society United Parkinson’s Disease Rating Scale Motor Subscale (MDS-UPDRS III) total score and gait and tremor sub-scores to improve following 8 weeks of Dance for PD® classes yet found no change in balance, while Bearrs et al. [20] found improvements in balance and functional mobility after 12 weeks. Ventura and colleagues [11] showed positive within group effect sizes for several motor outcomes, with the strongest effects noted for gait speed, and McNeely and colleagues [18] found adapted tango to improve motor symptom severity and functional mobility to a greater degree than Dance for PD®. Other studies have investigated the Dance for PD® model’s effects on aspects of cognition, emotional wellbeing [11, 21], and self-efficacy [19], demonstrating positive effects.

Recent systematic reviews on the topic of dance and Parkinson’s have highlighted the need for research to determine the effects of different dance styles, programs, and intensities [22] and have revealed that there have been few comparative trials and no randomized controlled trials (RCTs) evaluating dance classes modeled after the Dance for PD® method [23, 24]. The aforementioned studies investigating this program provide a strong foundation for future research yet they focused on Dance for PD®’s effects on motor, non-motor, and QOL-related outcomes, rather than outcomes relevant to the feasibility of future trials. Given that all but one of the existing studies reported small sample sizes as limitations and called for larger RCTs to confirm the generalizability and strength of effects, feasibility studies that are more descriptive and explicitly aim to determine if and how a full RCT could be carried out [25] are warranted.

## Study aims and objectives

The primary objective of this study was to assess the feasibility and acceptability of implementing dance classes based on the Dance for PD® model in Northern Ireland and conducting a trial investigating their effects. Feasibility and acceptability were measured in terms of recruitment (i.e., quotas met), retention (i.e., attendance rates and reported plans to continue dancing), outcome measures, and adverse events.

The secondary objectives of this study were to conduct preliminary analyses of the effects of dance on QOL and motor and non-motor characteristics in people living with Parkinson’s and to assess the appropriateness of these outcome measures for a larger, single-blind RCT.

## Methods

### Patient and public involvement

To ensure that the design and content of the tested trial would be meaningful and important to key potential stakeholders—i.e., people living with Parkinson’s and those close to them—patient and public involvement (PPI) activities were carried out during the design phase with the support of the Parkinson’s UK Research Involvement team. First, a survey was disseminated to the Parkinson’s UK Research Support Network. Of the 329 people who responded to the survey, 282 were living with Parkinson’s (age range 30–80+, time since diagnosis 2–20+ years, male/female/other/no answer 120/159/1/2); 41 identified as carers, partners, family members, or friends; and 6 were bereaved carers, partners, family members, or friends. The survey sought to understand if the aims of the research were clear, whether or not this area of research is seen as important by people affected by Parkinson’s, and how likely people would be to take part or recommend someone they know take part in the study. Responses revealed 97.9% of people living with Parkinson’s who took part in the survey found the aims of the research very or somewhat clear, 98.5% found the study to be very or somewhat important, and 79.4% indicated that they would be very or somewhat likely to participate, which supported the feasibility of such a trial. The survey also explored barriers to participation, the feasibility and selection of outcome measures, and ideas for capturing the experience of dancing (see Table 1).

**Table 1:** Details of responses of people living with Parkinson’s (*n* = 282) to PPI survey

Q. Do you think people would be more willing to participate if the study involved taking [dance] classes once or twice per week?	Once per week (141, 50%)
	Twice per week (41, 14.5%)
	Either (100, 35.5%)
Q. What would you expect to be the main barriers to participation in this research? Please select up to 2 options.	Transportation (150, 53.2%)
	Scheduling conflicts (137, 48.5%)
	Work/employment (40, 14.2%)
	Symptoms (51, 18.1%)
	I wouldn't expect there to be any barriers (29, 10.3%)
	Lack of interest (18, 6.4%)
	Stress imposed on carer (9, 3.2%)
	Other (34, 12.1%) Barriers specified (paraphrased): Location (14), Fear, embarrassment, shyness (5), Perceptions of dance (4), Other commitments (3), Cost (2), Lack confidence (2), Stress (1), No partner (1), Gender (1), Motivation (1), Group assignment (1)
Q. Before and after the 8-12 week intervention, participants […] will complete a series of physical and cognitive tests at the university. These would take a total of 90-120 minutes. Does this seem…	Too long (45, 16.0%)
	About right (172, 61.0%)
	I don't know (62, 22.0%)
	Too short (3, 1.1%)
Q. What outcome measures do you think would be most relevant and important to participants? Please select up to 3 that you think would be most important.	Balance (192, 68.1%)
	Walking ability (144, 51.1%)
	Cognitive abilities (113, 40.1%)
	Motor symptom severity (102, 36.2%)
	Mood (94, 33.3%)
	Endurance (61, 30.5%)
	Non-motor experiences of daily living (39, 13.8%)
	Motor experiences of daily living (71, 25.2%)
	None are relevant (1, 0.3%)
	Other (72, 25.5%) Outcomes suggested (paraphrased): Social benefits/impact (14), All (6), Posture (4), Self-worth/esteem, achievement (4), Medication (3), Sleep (3), Wellbeing (2), Coordination (2), Enjoyment of classes (1), Pain (1), Speech (1), QOL (1), Dexterity (1), Rigidity/flexibility (1), Self-management (1), Motivation (1), Independence (1)
Q. In this study, do you think we should assess participants when they are taking their medication as usual, or should we ask them to withhold their medication for 12 hours prior to the assessments?	On medication (211, 74.8%)
	Off medication, (0, 0%)
	No preference (71, 25.2%)
Q. As well as capturing standard outcome measures, we want to understand people's experiences of the programmes. What would be the best way of capturing this?	Face-to-face interview (85, 30.1%)
	Diary (62, 22.0%)
	Phone interview (57, 20.2%)
	Survey (31, 11.0%)
	Focus group (28, 9.9%)
	Other (19, 6.7%) Suggestions (paraphrased): Options, combination, or all of the above (10), Email (1), Video call (1), Diary with specific questions (1)

Data presented is the number of responses and percentage of 282 survey participants (no., %). Other suggestions/comments have been paraphrased and organized thematically for brevity; unclear or irrelevant comments were not included in the table

Following the survey, a discussion group was held at Queen’s University Belfast to discuss issues and ideas that arose from the survey with local people in greater detail. Twelve people attended the discussion group: eight people living with Parkinson’s, two partners/carers, one bereaved carer, and a dance instructor leading classes for people living with Parkinson’s in the community. The meeting was facilitated by the authors (AMC, MR, and MD) and the Parkinson’s UK Research Involvement Manager. Consent was sought to record the discussions using tape recorders and notetaking. As the data was not being analyzed as qualitative research data, the audio recordings were not transcribed verbatim [26], rather they were listened to again for further details. Discussion topics included advertising the study, encouraging participation, logistics, and outcome measures (i.e., duration of assessments, sensitive subjects, and capturing individual experiences). Following the meeting, key discussion points were fed back to those who attended and all stakeholders were invited to reply with any thoughts or questions that had come up since the meeting.

The information gathered as a part of the survey and the discussion group informed the design of the trial. This included recruitment strategies (e.g., visiting local support groups), the selection of outcome measures, and logistics relevant to the delivery of dance classes (e.g., venue selection, scheduling of class times) and assessment sessions (e.g., duration, information to be provided in advance). To confirm that the advice gathered through the PPI activities was satisfactorily implemented, advertising materials, participant information sheets, consent forms, outcome measures, and a summary of findings were reviewed by PPI volunteers living with Parkinson’s to ensure clarity and appropriateness.

### Participants

Participants were recruited from Queen’s University Belfast School of Psychology contacts, Parkinson’s UK recruitment channels, and the local community, beginning in Summer 2018. The original experimental protocol aimed to include a dance group and a control group that was to take part in a traditional exercise program in fall prevention and strength and balance training for comparison; 15 participants were to be randomized to either group using a random number generator. We used G*Power 3.1 to estimate a sample size of 10 participants living with Parkinson’s per group, with the Movement Disorder Society United Parkinson’s Disease Rating Scale Motor Examination (MDS-UPDRS III) as the primary outcome of interest and the alpha level set at 0.5 and power at 0.8. We aimed to recruit 15 participants living with Parkinson’s per group to account for dropouts.

Limited enrollment and time constraints forced a change in the protocol to a quasi-randomized controlled trial, with plans to assign participants sequentially first to the dance group and subsequently to the control group. This decision was made to reduce the amount of time participants would have to wait for the intervention to begin. Additionally, one of the intervention goals was to create community and five or fewer people in the classes would likely not have created the desired social atmosphere. Ultimately, by Spring 2019, only 10 eligible participants living with Parkinson’s in total had approached the research team with an interest in taking part making a control group infeasible in this setting at this time. The original protocol also aimed to recruit older adults without a diagnosis of Parkinson’s for comparison; however, recruitment for this group posed even more of a challenge with only four older adults living without Parkinson’s approaching the research team with an interest in taking part.

Participants were eligible for inclusion in the study if they had a diagnosis of Parkinson’s, reported benefit from anti-Parkinson medication, could walk 3 m with or without assistance, could stand for at least 30 min, and were able to provide informed consent. People were excluded if they had any major surgeries affecting movement in the past year, major injuries affecting movement in the past 6 months, a diagnosis of dementia, or serious neurological problems apart from Parkinson’s. Participants were instructed to continue with their regular medication regimens and exercise routines throughout the trial, and a diary was used to monitor any changes. Participants provided contact details for their GPs, who were informed of their participation in the trial via post. The study was reviewed and approved by the North East—Newcastle & North Tyneside 2 Research Ethics Committee within the UK Health Departments’ Research Ethics Service. All participants provided written informed consent prior to participation.

### Dance classes

Participants took part in 20 1-h dance classes over the course of 12 weeks, an average of two classes per week. The dance classes were modeled after training received in the Dance for PD® method, a program developed by Mark Morris Dance group in collaboration with the Brooklyn Parkinson’s Group [15]. Classes were held in person and in a group setting. The TiDIER (Template for Intervention Description and Replication) checklist was used to aid the description of the dance program in this report [27] (see Additional file 1).

#### Dance instructor and volunteers

The Dance for PD® program is built upon the principle that professionally trained dancers are movement experts with knowledge of balance, coordination, rhythm, and esthetic awareness that may be of value to people living with Parkinson’s [15, 28]. The dance classes were led by one instructor, the first author, a female ballet dancer with 10 years of experience performing professionally and teaching ballet to diverse age groups. At the start of the intervention, she had attended the Introductory and Advanced Dance for PD® Training Workshops in Toronto, Canada in 2016 and 2017, respectively, yet was not a certified Dance for PD® instructor [29]. She also had one year’s experience volunteering at a certified Dance for PD® class and one year’s experience leading such classes in the community. The instructor additionally underwent training to become a certified First Aider prior to commencing the classes.

Students from the School of Psychology and local dance artists also attended the classes, dancing with study participants, providing support to anyone who felt they needed it during standing exercises, and contributing to the overall social atmosphere. The volunteers attended a training session led by the first author that provided information about what Parkinson’s is and how it affects people, the class structure and content, and the role of a volunteer. The dance experience of the volunteers ranged from formal training or professional dance experience to no formal dance experience.

#### Class content and structure

The class content was developed from the instructor’s training received at the Introductory and Advanced Dance for PD® Training Workshops [30]. The intervention aimed to do the following: instill confidence in participants, create a community, stimulate mind-body connections, encourage creativity through improvisation and telling stories through movement, be joyful, and teach participants how to use imagery, rhythm, and attention to guide movement [15, 16]. The classes followed the three-part structure of the Dance for PD® program [15] (see Additional file 2 for example exercises). All classes began with a 20-min seated warm up consisting of exercises that aimed to warm, stretch, and coordinate the body to prepare for standing combinations. This was followed by 10–20 min at the barre during which time attention was focused on finding balance, maintaining placement and posture, and practicing weight shifts to provide participants with a supportive transition from sitting to standing. The final 20–30 min of class were spent “in the center” during which time participants traveled across the floor and danced with a partner or as a group. During this portion of class, there were opportunities both to learn choreography and to improvise. The classes were a tailored version of the Dance for PD® model in that the instructor drew from her own dance skills, knowledge, and experiences and also responded to the ability and interests (e.g., musical tastes) of the group. The instructor used dance terminology and discussed the significance of music and choreography throughout the classes to enhance learning.

#### Environment

The classes were held at the University’s Physical Education Centre in a fitness studio with wooden floors, a ballet barre, and full length mirrors along one wall that remained visible during classes. The venue had an atmosphere of a gym (sometimes noise from the weight room could be heard from next door during class), rather than an artistic dance space, but was selected because of its accessibility via public transport and ample, free parking, both of which were deemed important by PPI discussion group participants. The classes being on campus also made it easier for university students to be involved in the project.

### Assessments

Participants were tested before and after the 12-week dance intervention “ON” medication at a self-determined optimal time to control for medication-induced fluctuations. Baseline evaluations were conducted within 10 days prior to each participant’s first dance lesson. All assessments took place at a laboratory on campus at Queen’s University Belfast.

The pre-intervention evaluation (pre-test) included the following assessments to assess Parkinson’s motor symptoms, functional mobility, and balance: MDS-UPDRS III [31], Timed Up and Go (TUG) [32] & Dual-Task Timed Up and Go (DT-TUG) [33, 34], and the Sensory Organization Test (SOT) assessed using a NeuroCom Smart Balance Master (NeuroCom International, Clackamas, OR). The MDS-UPDRS III, TUG, and DT-TUG were videotaped and re-rated for accuracy by a second assessor blinded to the timepoint. Participants also completed the following assessments to assess aspects of cognition: Montreal Cognitive Assessment (MoCA) [35, 36], Trail Making Tests A & B (Trails A&B) [37], and Digit Symbol Substitution Test (DSST) and Digit Span (DS) Forwards and Backwards (subtests of the Wechsler Adult Intelligence Scale-III). Furthermore, participants completed four questionnaires to assess health-related QOL, experiences of freezing of gait, symptoms of depression, and fear of falling, respectively: the Parkinson’s Disease Questionnaire (PDQ-39) [38], Freezing of Gait Questionnaire (FOG-Q) [39], Patient Health Questionnaire-9 (PHQ-9) [40, 41], and Falls Efficacy Scale International (FES-I) [42, 43]. Proprioceptive acuity in the ankle joint was also measured using a joint position matching task [44]; this outcome was excluded from the analysis due to a measurement error but has been included in the list of outcomes to provide a complete description of the evaluation session.

The post-intervention evaluation (post-test) exactly mirrored the pre-test and took place within one week after completion of the twentieth dance class. At post-test, participants also completed an Exit Questionnaire adapted from Hackney et al. [12] that evaluated their experiences in the program and perceived benefits of dancing (see Additional file 3). Participants were offered £20 at the completion of the study to cover the cost of transport to and from assessment sessions.

### Data analysis

Recruitment was measured as percentage recruited out of target (30 participants living with Parkinson’s). Retention was measured as the percentage of people who completed the study and attendance was calculated as the percentage of classes completed by all who commenced the intervention. Adverse events reported in exercise diaries or observed by or communicated to the dance instructor in the dance classes were tabulated and synthesized qualitatively.

Changes in outcome measures from pre- to post-test were analyzed using JASP (Version 0.13.1) [45]. If difference scores were normally distributed, a paired-samples *t* test was used and if they were not, a Wilcoxon signed-rank non-parametric test was used. Alpha level in both cases was set at α = 0.05. As statistical analyses were carried out separately for distinct measures, corrections for multiple analyses were not applied. Effect sizes were calculated using Cohen’s *d* if the data were parametric or a rank-biserial correlation if non-parametric. Per protocol analysis was used. The median and interquartile ranges were calculated for responses to each item on the Exit Questionnaire.

## Results

### Feasibility and acceptability

**Fig. 1:**
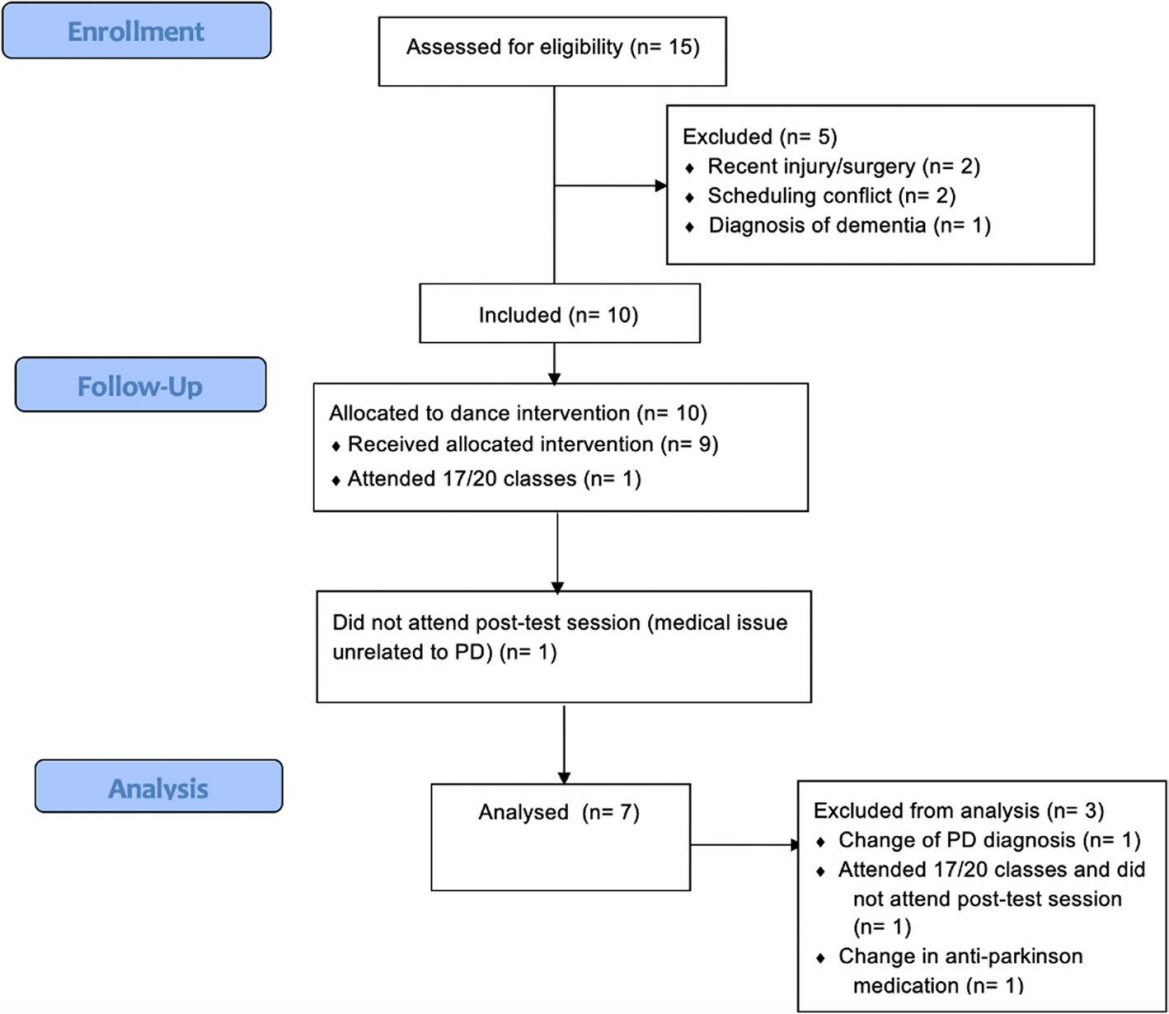
CONSORT participant flow diagram. The number of participants approached is not included in this diagram because, due to a primary strategy of disseminating information about the study in the community, it is not possible to report the number of prospective participants reached during the recruitment stage

Only ten of 30 participants living with Parkinson’s (33%) were recruited from University and Parkinson’s UK contacts and the community (see Fig. 1 and Table 2). Four adults who were not living with Parkinson’s also took part in the dance classes, including two partners of participants, a friend of a participant, and a bereaved carer; they completed 20 sessions and contributed to the overall social atmosphere.

**Table 2:** Characteristics of participants

Participant	Age, years	Sex	Education, years	PD duration, years	H&Y	Medications
1	70	F	18	3	2	C/L
2	67	M	18	5	1	C/L, Ras, Rop
3	66	M	18	9	1.5	C/L, Ras, Rop
4	53	M	12	3	2.5	C/L
5	72	M	16	1	1	C/L, Rot.
6	72	M	19	2	2	C/L
7	68	M	16	5	1.5	C/L, Ras, Rop

*PD* Parkinson’s disease, *H*&*Y* modified Hoehn and Yahr Stage, *C*/*L* Carbidopa/levodopa, *Ras*. Rasagiline, *Rop*. Ropinirole, *Rot*. Rotigotine

Retention on the contrary was extremely high with all but one participant completing 20 classes within 12 weeks and the post-test assessments. The participant who did not complete the intervention attended 17 of the 20 required classes, making the attendance rate 98.5%.

Participation in the exercise diary was moderate, with all but two participants returning it to the research team for review at the post-test session. One of the eight participants who returned the diary only had entries for six of the twelve weeks they attended classes. At the post-test session, participants were asked if they had continued with their usual routines outside of class; all reported that they had, and the entries in the exercise diaries supported this qualitatively.

### Adverse events and effects

There were no adverse events (e.g., falls, injuries) during the dance classes. However, two participants reported in their diaries feeling soreness and pain in legs, shoulders, arms, and wrists during and immediately after the dance classes. Two participants reported soreness and pain outside of and not attributed to the dance classes, three experienced no soreness/pain at all during the course of the program, and one reported that stiffness and soreness was alleviated by the dance classes. Outside of the dance classes, one participant experienced two non-injurious falls and one experienced a non-injurious fall and a near miss.

### Outcome measures

Of the eight dimensions of the PDQ-39 (Mobility, Activities of Daily Living, Emotional Well-being, Stigma, Social Support, Cognition, Communication, and Bodily Discomfort), a significant median decrease (a lower score indicates improvement) was found for the Bodily Discomfort dimension only. No statistically significant differences were found for the other seven dimensions or summary index (SI) (see Table 3).

**Table 3:** Results for Parkinson’s Disease Quality of Life Scale (PDQ-39)

Dimension	Pre-test	Post-test	*P*	Effect size
Mobility	37.50 ± 40.00 (2.50, 77.50)	25.00 ± 30.00 (2.50, 67.50)	0.058	− 1.000
ADLs	41.67 ± 41.67 (16.67, 87.50)	33.33 ± 37.50 (8.33, 95.83)	0.235	− 0.536
Emotional Well− Being	29.17 ± 29.17 (4.17, 45.83)	12.50 ± 20.83 (4.17, 66.67)	0.410	− 0.467
Stigma	12.50 ± 18.75 (0, 43.75)	12.50 ± 12.50 (0, 62.50)	0.890	− 0.133
Social Support	8.33 ± 25.00 (0, 41.67)	0 ± 33.33 (0, 41.67)	0.423	− 0.667
Cognition	31.25 ± 31.25 (12.50, 68.75)	18.75 ± 6.25 (18.75, 68.75)	0.170	− 0.733
Communication	25.00 ± 25.00 (8.33, 50.00)	16.67 ± 16.67 (0, 41.67)	0.396	− 0.393
**Bodily Discomfort**	**41.67** ± **33.33 (33.33, 75.00)**	**25.00** ± **50.00 (8.33, 66.67)**	**0.036**	− **1.000**
Summary Index	26.56 ± 20.63 (11.25, 57.08)	16.25 ± 19.38 (10.21, 63.91)	0.078	− 0.786

Wilcoxon signed-rank test. Scores reported are medians and interquartile ranges. A decrease in score indicates improvement. Effect size reported is the Rank-biserial correlation

For motor outcomes (Table 4), the difference between pre-test values and post-test values for the MDS-UPDRS III was not significant, nor was the difference between pre-test values and post-test values for the SOT Composite Score. Additionally, there were no statistically significant differences pre- and post-intervention for any of the six equilibrium scores. All of the seven participants included in the analysis demonstrated an improvement in score on the TUG (i.e., the time to complete the task was less at post-test than pre-test), and there was a statistically significant median decrease from pre-intervention to post-intervention. In the DT-TUG, there were no significant differences pre-intervention to post-intervention. In questionnaires concerning motor symptoms (Table 4), no statistical significance was found for the FOG-Q nor the FES-I between pre-test and post-test.

**Table 4:** Results for motor outcomes

Outcome	Pre-test	Post-test	Change score	*P*	Effect size
MDS-UPDRS-III^a^	28.57 (14.36)	23.71 (10.52)	− 4.86	0.072	− 0.823
**TUG (sec)**^**b**^	**11.18 (2.10)**	**9.48 (1.27)**	− **1.70**	**0.016**	− **1.000**
DT-TUG (sec)^b^	12.48 (2.32)	11.76 (2.67)	− 0.72	0.327	− 0.429
FOG-Q^b^	5.14 (2.80)	4.86 (3.39)	− 0.28	0.589	− 0.286
FES-I^b^	31.71 (11.83)	30.71 (11.38)	− 1.00	0.833	− 0.143
SOT^a^	69.71 (5.94)	73.00 (4.40)	3.29	0.236	0.498
SOT-E1^a^	93.38 (1.08)	92.38 (2.70)	− 1.00	0.180	− 0.574
SOT-E2^a^	90.76 (2.46)	90.38 (1.86)	− 0.38	0.740	− 0.131
SOT-E3^a^	90.71 (3.47)	90.10 (3.43)	− 0.61	0.644	− 0.184
SOT-E4^a^	73.52 (7.20)	78.67 (5.77)	5.15	0.116	0.695
SOT-E5^a^	49.48 (21.13)	54.33 (9.56)	4.85	0.446	0.308
SOT-E6^a^	46.48 (13.32)	56.52 (15.14)	10.04	0.290	0.439

Pre- and post-test values presented are means and standard deviations

*MDS-UPDRS-III* Movement Disorder Society United Parkinson’s Disease Rating Scale Part III, *TUG* Timed Up and Go Test, *DT-TUG* Dual-Task Timed Up and Go (cognitive), *FOG*-*Q* Freezing of Gait Questionnaire, *FES*-*I* Falls Efficacy Scale International, *SOT* Sensory Organization Test Composite Score, *E1* eyes open firm surface, *E2* eyes closed on firm surface, *E3* eyes open with sway referenced visual surround, *E4* eyes open on sway referenced support surface, *E5* eyes closed on sway referenced support surface, *E6* eyes open on sway referenced support surface and surround

^a^Data were analyzed using Student’s *t* test. Effect size reported is Cohen’s *d*

^b^Data were analyzed using the Wilcoxon Signed Rank test. Effect size reported is the Rank-biserial correlation

With regards to non-motor outcomes (Table 5), there was a significant median decrease (a lower score indicates improvement) in the PHQ-9 score from pre- to post-intervention. No significant differences between pre- and post-test data were found for outcomes measuring aspects of cognition.

**Table 5:** Results for non-motor outcomes

Outcome	Pre-test	Post-test	Change score	*P*	Effect size
**PHQ-9**	**7.71 (5.62)**	**5.71 (5.74)**	− **2.00**	**0.034**	− **1.000**
MoCA	26.71 (1.89)	27.43 (1.51)	0.72	0.671	− 0.238
Trail A (sec)	49.91 (12.69)	43.90 (14.24)	− 6.01	0.219	0.571
Trail B (sec)	92.47 (23.12)	99.76 (32.04)	7.29	0.578	− 0.286
DS Forward	11.14 (2.55)	11.43 (1.81)	0.29	0.798	− 0.143
DS Backward	6.43 (3.55)	7.00 (2.94)	0.57	0.526	− 0.333
DS Total	17.57 (4.32)	18.43 (4.58)	0.86	0.400	− 0.429
DSST	50.71 (11.90)	51.43 (12.38)	0.72	0.865	− 0.107

Non-motor outcomes were analyzed using the Wilcoxon-signed rank test. Pre- and post-test values presented are means and standard deviations. Effect size reported is the Rank-biserial correlation

*PHQ-9* Patient Health Questionnaire 9, *MoCA* Montreal Cognitive Assessment, *Trail A* Trail Making Test Part A, *Trail B* Trail Making Test Part B, *DS* Digit Span, *DSST* Digit Symbol Substitution Test

### Exit questionnaire

Findings from the Exit Questionnaire revealed that participants enjoyed the classes and would continue with them if they were offered in the community. Participants additionally reported perceiving moderate improvements in several aspects of physical and emotional health (see Table 6).

**Table 6:** Participant responses to Exit Questionnaire

Questionnaire Item	Median (1st, 3rd quartiles)
I enjoyed the program	1 (1.00, 1.00)
Balance improved	2 (2.00, 2.50)
Walking improved	3 (2.00, 3.00)
Coordination improved	2 (2.00, 2.50)
Strength improved	2 (1.00, 2.50)
Flexibility improved	2 (1.50, 2.50)
Mood improved	2 (2.00, 2.00)
Aches/pains improved	3 (1.50, 3.00)
I would continue	1 (1.00, 1.50)
I use ideas/skills learned in class in ADLs	2 (1.50, 2.00)

Participants rated each item on the Exit Questionnaire on a scale of one to five. 1, Strongly agree, 2, Somewhat agree, 3, Neither agree nor disagree, 4, Somewhat disagree, 5, Strongly disagree

*ADLs* Activities of daily living

Open-ended questions invited participants to share what they liked and disliked most about the program and if they experienced any benefits not highlighted in the questionnaire. In describing what they liked most, participants cited “the company” and “meeting new people,” reporting that the social aspect of the classes was motivating and encouraged them to “get out.” Participating in the research created a “common cause” and being in the company of other people living with Parkinson’s fostered a “sense of belonging” making people feel “part of the group rather than the outcast.” The dancers and student volunteers were described as “wonderful,” “brilliant,” “helpful,” and “respectful.” Participants also enjoyed learning about the variety of music and dance styles and steps taught in class, and one said the teacher “communicated enthusiasm and skill.” Reported dislikes included thinking the program would be more strenuous, feeling uncomfortable dancing with other people during partnered exercises, or disliking improvisation because “it puts you out there” and “makes you very vulnerable.” There was also one comment that the room was too cold. Some participants said there was not anything that they disliked about the dance classes.

When asked about perceived improvements not covered in the Exit Questionnaire, improvements in mood or mental state were cited most frequently:

“I felt my mood has improved. I think because the movement was done to music, I think. I like listening to lots of types of music.”

“Felt better in your head.”

“Mood – 100% after leaving each week. It was great fun.”

“Peace of mind. I felt more content with life when I came out of it because I enjoyed it so much. There was a great atmosphere about the room, the social aspect.”

“The entire mood of the class has been uplifting. There has been much laughter – often instigated by the teacher. I never leave the class in anything but a happy mood.”

Benefits of dancing described included disappearance of upper body stiffness, increased energy that led to walking more on class days, slight improvements in coordination and balance, improved strength and mental ability, and increased confidence.

Some participants mentioned that some class material highlighted “physical deficits and mobility problems,” such as declining sense of rhythm or coordination and inflexibility. A few described working to correct these issues with greater awareness outside of class. For example, several noted that they became more aware of their posture and now make efforts to correct it. Some participants also described using tools learned in class in everyday life. For example, one participant reported using breathing exercises when anxious or fatigued, and another reported using the warm-up exercises outside of class to “stop the stiffness” and “feel better as a result of that.”

## Discussion

This study highlighted the challenges associated with the feasibility of running a larger trial investigating dance for people living with Parkinson’s in Northern Ireland due to recruitment issues. At the same time, this study also demonstrated the acceptability of implementing a dance program for people living with Parkinson’s in this setting and findings suggested that participants were motivated to continue dancing with high levels of adherence. The results also suggest that dance may improve functional mobility, symptoms of depression, and bodily discomfort in people living with Parkinson’s as the people who took part demonstrated improved scores on these outcomes. The dance program was also reported to provide social support and lead to an improved mood, with positive and negative bodily experiences reported. The outcome measures were deemed feasible, with some considerations proposed for future trials.

### Feasibility and acceptability

A primary constraint on feasibility identified during this study was the challenge of recruiting participants. The study originally aimed to include a control group that was to practice a traditional physiotherapy-designed exercise program for comparison; however, it became clear in Spring 2019 that this would not be possible when no further people approached the research team with an interest in participating in the study. Poor recruitment remains the leading reason for the discontinuation of RCTs, and reports of these trials are less likely to be published [46]. Recruitment has also been an ongoing challenge in this research area, with the first study to investigate the impact of dancing in Parkinson’s citing delays due to difficulty identifying suitable participants and lack of support from local physicians [47]. The authors suspected that this was due to the “non-traditional” nature of the intervention and noted that contacting prospective participants via support groups yielded better results. Many systematic reviews summarizing the results of subsequent studies investigating dance and Parkinson’s also note small sample sizes as limitations [22–24, 48, 49].

Our primary recruitment strategies were disseminating information about the study via Parkinson’s UK’s Research Support Network (RSN), dedicated webpage, and digital channels and through visiting local support groups. We also sent information to the local chapters of other organizations, such as the University of the Third Age, to disseminate to their members. As a part of our PPI strategy, a survey was conducted prior to commencing the research, and it revealed that the majority of people living with Parkinson’s who participated in the survey found the aims of the research clear, deemed the study to be important, and indicated that they would be likely to participate (see PPI details in “Methods” section). This was encouraging, yet it was realized later that these findings may not have been representative of all regions of the UK, particularly the demographic from which we were recruiting. Parkinson’s UK’s RSN grew to over 5000 members by the end of 2018 [50], but only 50 of these members were living in Northern Ireland when recruitment efforts officially ended in Spring 2019 (Email correspondence, 1 May 2019). This represented just over 1% of the estimated 3716 people living with Parkinson’s in Northern Ireland [51].

The low engagement in Parkinson’s UK’s RSN in Northern Ireland may indicate a lack of awareness, interest, or trust in research and its processes in this community, which may have impacted recruitment efforts. This highlights the importance of a clear understanding of the community during the research design stage and communication among stakeholders. An increasing amount of research activity and visibility in the region may be a solution to this issue. In addition to barriers in research, there may also be barriers to dancing for a person living with Parkinson’s, including accessibility, the assumption that dance is a young or highly mobile person’s activity, or the confrontation of spending time with other people living with more advanced stages of the condition [52]. Survey participants also reported barriers related to accessibility and perceptions of dance, as well as fear, embarrassment, and lack of confidence. Both healthcare and arts practices like dance are relational (i.e., the environment influences the outcome), esthetic (i.e., people have preconceived notions about health and dance), and temporal (i.e., both health and art occur within the context of lived experience) [53]; therefore, more consideration of context may be needed when determining how to most effectively engage participants in this research area. Given the widespread implementation success of community-based dance programs for people living with Parkinson’s, networked research efforts across institutions and regions should be considered and piloted for future RCTs.

Even though recruitment quotas were not met, the acceptability of the intervention was clear among those who took part with the attendance rate at 98.5%. One of the 10 participants attended 17 out of the 20 classes and was the only participant to not complete the intervention or post-test session. Previous research has suggested that dance may be motivating and lead to higher adherence rates than other physical activity programs [12, 13], which is supported by the retention rates in this trial. More than 24 classes were offered to accommodate rolling enrollment in the study, and six participants (43%), five living with Parkinson’s and one partner without Parkinson’s, took part in these additional sessions after completing the assigned 20 classes and their post-test assessments. The “drop-out” in this study also returned to the community-based classes after the research was complete, and five of the 10 participants (50%) were still dancing 2 years later (unpublished observations), further supporting the acceptability of the program and the participants’ motivation to continue dancing. Of course, it must also be considered that self-selection bias played a role in the acceptability of the intervention, especially given the issues with recruitment.

### Outcome measures

The PDQ-39 SI, a global measure of Parkinson’s impact on QOL, did not significantly improve following the dance intervention; however, the mean change in score (*d* = − 6.369) reached the threshold of a minimally important difference (MID) (− 4.72) [54]. The impact of these types of dance classes on QOL has been variable [13, 17, 18, 20, 21], suggesting either that programs are delivered inconsistently or the outcome measures evaluating QOL are not sensitive enough. The dimension of the PDQ-39 that measures the impact of bodily discomfort (e.g., “aches and pains in joints or body”) on QOL showed a significant change and the mean change in score (*d* = − 14.29) was much larger than the MID (− 2.1) [55]. While the effects of dance on discomfort and pain in people living with Parkinson’s have yet to be explored, dance has been shown to refocus awareness from painful to pleasurable bodily feelings [56] and relieve pain in other clinical groups [57, 58]. This is worth investigating further as pain is among the symptoms most associated with poor QOL in Parkinson’s [9, 10]. Researchers should also consider that the MIDs vary across dimensions of the PDQ-39 and therefore should select the MID of the dimension of primary interest, whether it be the SI score or otherwise, to calculate the sample size for future trials [54, 55].

Prior research has demonstrated that Dance for PD® style classes may improve balance in people living with Parkinson’s [20]; however, other dance programs have been suggested to be more effective at training balance given that Dance for PD® classes involve spending a significant portion of class time seated [13, 18]. In this study, no significant changes were found in balance, measured using the SOT. The TUG conversely demonstrated significant change; however, no participant experienced an MID (3.5 s) [59] in their score with the average being a decrease of 1.70 s. Changes in motor symptom severity and freezing of gait were also evaluated using the MDS-UPDRS III and FOG-Q, respectively, and no improvements were found in either outcome. The mean change in the MDS-UPDRS III (− 4.68) did meet the threshold of an MID and no participants experienced a clinically pertinent worsening [60]. While several trials investigating Dance for PD® classes have demonstrated improvements in these outcomes, results have again been variable [13, 18]; inconsistencies have also been observed in the investigations of other dance interventions [61]. More research is needed to determine whether particular dance styles or intensity levels are needed to have an impact on motor complications of Parkinson’s and to confirm when a measured change is truly “significant” to people living with Parkinson’s.

Symptoms of depression as measured by the PHQ-9 reduced significantly over the course of the program. The PHQ-9 was selected because it is less burdensome than other depressive scales, a self-rating tool in the public domain [62] that has been validated as a screening measure for major depression in Parkinson’s [41, 63], and extensively used in clinical research studies [64]. The Geriatric Depressive Scale-30, however, is likely the better choice for a future, larger trial as it has been shown to be more sensitive than the PHQ-9 and it avoids somatic symptoms of depression that overlap with symptoms of Parkinson’s [65]. In contrast to Ventura et al. [11] and Kalyani et al. [21], the dance intervention had no effects on cognitive outcomes in this study. Recent systematic reviews on dance and Parkinson’s have also found assessments of cognition to be limited [23] or to result in inconsistent directions of effects depending on the aspect of cognition being measured [61], further highlighting the need for more research in this area.

#### Participant experience

Many participants emphasized the impact that the classes’ positive atmosphere had on their moods, saying that they felt “more content with life” and “better in your head.” Future trials may consider including semi-structured interviews or focus groups to gain better understandings of how the elements of dance mentioned, including music, laughter, energy, and “the social aspect,” may contribute to improved mental and emotional states. The inclusion of qualitative data would enrich the understanding of any effects of dance classes experienced by people living with Parkinson’s, help guide the selection of quantitative measures intended to demonstrate such effects, and further our understanding of complex or ambiguous aspects of dancing that are difficult to capture quantitatively. Future research might also consider measuring the impact of dancing on happiness, which has been suggested to measure an intervention’s value beyond what is captured by measuring motor aspects of Parkinson’s and QOL [66], or perhaps joy, which is more often related to connecting with others than happiness [67].

Another common theme in responses to the Exit Questionnaire was “the company,” suggesting that the “sense of belonging” and the “social side of it” led people to continue participating in the program; the social importance of dance classes was also found in other similar studies [68, 69]. People living with Parkinson’s experience stigma and social isolation [8], and a sense of belonging is a core dimension of social inclusion for people living with disabilities and something that community rehabilitation programs should seek to address [70]. The group also requested that classes be followed by tea and coffee so they could get to know each other, since there were few opportunities to talk while dancing. Future trials may consider including a social dimension outside of the intervention from the start, given that this is commonplace in community programs and has been requested before in prior studies [12]. When participants were asked if there was “anything else about their experience that they would like to share,” many mentioned the dance artists and student volunteers who participated, referring to them as “wonderful,” “brilliant,” “helpful,” and “respectful,” suggesting that they were integral to creating a positive social atmosphere. Interestingly, when people living with Parkinson’s were asked about other important outcome measures not already listed by the researchers in the PPI survey, outcomes related to the social impact of dancing were most commonly recommended.

Negative feedback was also noted with some participants reporting pain/soreness while dancing. During the classes, participants were encouraged by the instructor to work within their own personal range of motion and none of the pain or soreness was described as lasting, so it is unclear if it was avoidable or harmful. Some participants also felt that some class material made them more aware of their physical deficits, such as posture, and encouraged them to correct these with greater awareness. This increased body awareness seemed to be presented as a positive in this context; however, postural complications of Parkinson’s are common and disabling [71], and it remains unclear if making participants aware of their posture has value and (if so) whether it should be framed in a particular way to avoid discouragement. This finding also calls attention to the importance of understanding more deeply the experience of dancing.

## Limitations

The main limitation of this study is the fact that we changed our study protocol in order to commence the intervention with the dance group and subsequently failed to recruit enough for our control group. This left us unable to complete our assessment of feasibility and compare the acceptance of the dance program to another intensity-matched exercise program; the lack of control group also prevents attributing any positive effects on outcomes seen in this study directly to the dance intervention itself. Moreover, our strategy of recruiting through the community left us with an unclear number of how many people living with Parkinson’s were actually reached in the recruitment stage. Given ongoing issues with small sample sizes and recruitment issues in this research area, future studies should aim to accurately record a response rate to paint a better picture of the number of people willing to participate in such research. Methods for managing self-selection bias should also be considered.

This study investigated the effects of dance in people living with Parkinson’s who could stand for 30 min and walk at least 3 m. This ensured that all participants could (a) complete the selected assessments and (b) practice the same exercises in the classes for consistency; however, it also meant that only people living with mild to moderate Parkinson’s could take part. The lack of diversity in disease severity is a limitation of this study and the broader dance and Parkinson’s literature [24]. This is an important avenue of future research, especially given that the Dance for PD® training program emphasizes inclusivity by encouraging instructors to provide adaptations and modifications for all exercises.

Another limitation of this study is that we did not formally control for anti-Parkinson medication-induced fluctuations. Participants took part in assessment sessions at a self-determined time when their medication was working well; however, this was not always at exactly the same time during the pre- and post-test assessments. Other studies of dance and Parkinson’s have found controlling for medication to be a challenge even when the assessment sessions took place at the same time at both time points [72]. Larger, future trials should consider how to optimally control for medication-induced fluctuations in order to strengthen the validity of results.

## Conclusions

This study highlighted the challenges associated with the feasibility of using an RCT design in this setting, demonstrated the acceptability of implementing a dance program inspired by the Dance for PD® model in Belfast, Northern Ireland for people living with Parkinson’s, and made suggestions for future research. The results support existing evidence demonstrating that dance may improve functional mobility and symptoms of depression in people living with mild to moderate Parkinson’s, though these findings should be carefully interpreted in the context of the study design and limitations. The small sample size limits the generalizability of the statistical results, though it was not among the aims of this study to test the efficacy of the intervention. The findings also support the idea that meeting and dancing with other people living with Parkinson’s is motivating and fosters a sense of belonging, and that dancing has the potential to support several aspects of physical, emotional, mental, and social health.

## Supplementary Information

**Additional file 1.** The TIDieR (Template for Intervention Description and Replication) Checklist.

**Additional file 2.** Example exercises.

**Additional file 3.** Exit Questionnaire.

## Data Availability

The datasets used and/or analyzed during the current study are available from the corresponding author on reasonable request.

## Abbreviations

QOL: Quality of life
QOLS: Oregon Health & Sciences University Quality of Life Scale
PDQ-39: Parkinson’s Disease Questionnaire
RCT: Randomized controlled trial
PPI: Patient and public involvement
MDS-UPDRS III: Movement Disorder Society United Parkinson’s Disease Rating Scale Motor Subscale
TUG: Timed Up and Go test
DT-TUG: Dual-task Timed Up and Go test
SOT: Sensory Organization Test
MoCA: Montreal Cognitive Assessment
Trails A&B: Trail Making Tests A and B
DSST: Digit Symbol Substitution Test
DS: Digit Span
FOG-Q: Freezing of Gait Questionnaire
PHQ-9: Patient Health Questionnaire
FES-I: Falls Efficacy Scale International
SI: Summary index
RSN: Research Support Network
MID: Minimally important difference
FAB: Fullerton Advanced Balance Scale
BBS: Berg Balance Scale
