# Supplementary material for: Evaluating the effects of dance on motor outcomes, non-motor outcomes, and quality of life in people living with Parkinson’s: a feasibility study
Source: Pilot Feasibility Stud. 2022 Feb 9;8:36. doi: 10.1186/s40814-022-00982-9 (PMC8827282; doi:10.1186/s40814-022-00982-9)
Supplement: Supplementary file 2 — Additional file 2. Example exercises. [file 40814_2022_982_MOESM2_ESM.docx]

**Additional File 2**

| **Example Exercise** | **Example music** | **Description** | **Aim** |
| --- | --- | --- | --- |
|  |  |  |  |
| ***Seated Exercises*** |  |  |  |
| Qi gong inspired warm up | Chopin, Nocturn in C Minor | Stretch arms to the ceiling, let the arms fall like silk to the sides; stretch one arm towards the ground and one to the ceiling, directing gaze towards upper or lower hand; pull an imaginary bow to release an arrow; stretch forward to relax on the legs; twisting and circling the torso | Warm up and increase range of motion in the upper body extremities and torso; stimulate mind-body connection through connecting breath and gaze to movement; explore  different movement qualities (strong, silk-like) |
| Rhythmic warm up | Traditional Spanish Flamenco music | Toe and heel tapping, marching, and stomping in various rhythms; circling of the hands and arms; playing castanets (articulating and playing with fingers) | Warm up and increase range of motion in lower body; using rhythm and attention to guide movement; introduce elements of folk or other cultural dance for inspiration |
| Storytelling through movement | Gershwin, I Got Rhythm | Act out scene inspired by American in Paris; learn elements of tap dancing (e.g., shuffle) | Practicing storytelling through movement; introduce information about dancers and choreography for inspiration; facilitate mobility in ankles and knees |
| Choreographic sequence | Bach, Cello Suite No. 3 | Learn an excerpt from the Bourree Project, an initiative where Dance for PD teachers around the world taught a modified version of Mark Morris choreography; practice sequence over the course of several weeks | Stimulate memory and attention through challenging participants to learn a choreographic sequence; instill confidence |
|  |  |  |  |
| ***Barre*** |  |  |  |
| Plie and relevé | Gershwin, Embraceable You | Knee bends; calf and toe raises; stretching arms, torso, and legs in various directions while standing; shifting weight from one leg to the other | Find balance, learn principles of posture and alignment, develop strength in lower legs |
| Rhythm exercise | Alunelu | Side stepping and stomping in a complex rhythmic pattern | Challenge balance; use rhythm, memory, and attention to guide movement; introduce elements of folk or other cultural dances; instill confidence through repetition |
| Tendu and adagio | Ella Fitzgerald, Blue Skies | Stretch foot along the floor until leg is fully extended to the front, side and back; repeat and extend until foot is lifted off the floor | Challenge balance; learn principles of alignment; strengthen legs; practice movement qualities (smooth, slow); instill confidence |
|  |  |  |  |
| ***Center*** |  |  |  |
| Rhythmic walking | Temptations, Ain’t Too Proud to Beg | Walk forwards (heel-toe and toe-heel) and sideways to music in various tempos and rhythmic patterns | Challenge balance; explore rhythms and modes of walking |
| Partnered dance | Andy Williams, Moon River | Step forwards, sideways, and backwards with partner in various rhythms (e.g., waltz) and patterns | Foster connection among participants; learn rhythms of various dance styles |
| Folk dance in a circle | Zorba's Dance | Standing all together in a circle, walk sideways, forwards, and backwards in varying tempos and patterns | Foster connection among participants; using rhythm and synchronization to guide movement;  introduce elements of folk or other cultural dance for inspiration |
| Mirroring improvisation | Bob Marley, Could You Be Loved | Stand or sit opposite a partner and guide or follow (i.e., mirror) as each takes turns improvising | Encourage and challenge creativity; use attention and focus to follow movement; instill confidence; foster connection |
| Pass the pulse | None | Hold hands in a circle and pass a 'pulse' (hand squeeze) from person to person and thank them for dancing | Foster connection and create sense of community; acknowledge everyone in class |
