# Supplementary material for: Evaluating the effects of dance on motor outcomes, non-motor outcomes, and quality of life in people living with Parkinson’s: a feasibility study
Source: Pilot Feasibility Stud. 2022 Feb 9;8:36. doi: 10.1186/s40814-022-00982-9 (PMC8827282; doi:10.1186/s40814-022-00982-9)
Supplement: Supplementary file 3 — Additional file 3. Exit Questionnaire. [file 40814_2022_982_MOESM3_ESM.docx]

Exit Questionnaire

For each of the following statements, please circle the opinion closest to your own.

1. I enjoyed participating in the dance/exercise classes.
   1. Strongly agree
   2. Somewhat agree
   3. Neither agree nor disagree
   4. Somewhat disagree
   5. Strongly disagree
2. My balance has improved since starting this programme.
   1. Strongly agree
   2. Somewhat agree
   3. Neither agree nor disagree
   4. Somewhat disagree
   5. Strongly disagree
3. My walking has improved since starting this programme.
   1. Strongly agree
   2. Somewhat agree
   3. Neither agree nor disagree
   4. Somewhat disagree
   5. Strongly disagree
4. My coordination has improved since starting this programme.
   1. Strongly agree
   2. Somewhat agree
   3. Neither agree nor disagree
   4. Somewhat disagree
   5. Strongly disagree
5. My strength has improved since starting this programme.
   1. Strongly agree
   2. Somewhat agree
   3. Neither agree nor disagree
   4. Somewhat disagree
   5. Strongly disagree
6. My flexibility has improved since starting this programme.
   1. Strongly agree
   2. Somewhat agree
   3. Neither agree nor disagree
   4. Somewhat disagree
   5. Strongly disagree
7. My mood has improved since starting this programme.
   1. Strongly agree
   2. Somewhat agree
   3. Neither agree nor disagree
   4. Somewhat disagree
   5. Strongly disagree
8. I feel I have less aches and pains since starting this programme.
   1. Strongly agree
   2. Somewhat agree
   3. Neither agree nor disagree
   4. Somewhat disagree
   5. Strongly disagree
9. I would continue with classes if offered.
   1. Strongly agree
   2. Somewhat agree
   3. Neither agree nor disagree
   4. Somewhat disagree
   5. Strongly disagree
10. I use ideas/skills learned in the classes in my daily life.
    1. Strongly agree
    2. Somewhat agree
    3. Neither agree nor disagree
    4. Somewhat disagree
    5. Strongly disagree

Open Ended Questions

1. What did you like most about the programme?
2. What did you like least about the programme?
3. Did you experience any improvements as a result of the programme that were not mentioned above?
4. Is there anything else about your experience in the programme that you would like to share?
